# Transcriptome profile of a bovine respiratory disease pathogen: *Mannheimia haemolytica *PHL213

**DOI:** 10.1186/1471-2105-13-S15-S4

**Published:** 2012-09-11

**Authors:** Joseph S Reddy, Ranjit Kumar, James M Watt, Mark L Lawrence, Shane C Burgess, Bindu Nanduri

**Affiliations:** 1 College of Veterinary Medicine, Mississippi State University, Mississippi State, MS 39762, USA; 2 Center for Clinical and Translational Science, University of Alabama at Birmingham, Birmingham, AL 35294, USA; 3 Eagle Applied Sciences LLC, San Antonio, TX 78248, USA; 4 College of Agriculture and Life Sciences, University of Arizona, Tucson, AZ 85721, USA

## Abstract

**Background:**

Computational methods for structural gene annotation have propelled gene discovery but face certain drawbacks with regards to prokaryotic genome annotation. Identification of transcriptional start sites, demarcating overlapping gene boundaries, and identifying regulatory elements such as small RNA are not accurate using these approaches. In this study, we re-visit the structural annotation of *Mannheimia haemolytica *PHL213, a bovine respiratory disease pathogen. *M. haemolytica *is one of the causative agents of bovine respiratory disease that results in about $3 billion annual losses to the cattle industry. We used RNA-Seq and analyzed the data using freely-available computational methods and resources. The aim was to identify previously unannotated regions of the genome using RNA-Seq based expression profile to complement the existing annotation of this pathogen.

**Results:**

Using the Illumina Genome Analyzer, we generated 9,055,826 reads (average length ~76 bp) and aligned them to the reference genome using Bowtie. The transcribed regions were analyzed using SAMTOOLS and custom Perl scripts in conjunction with BLAST searches and available gene annotation information. The single nucleotide resolution map enabled the identification of 14 novel protein coding regions as well as 44 potential novel sRNA. The basal transcription profile revealed that 2,506 of the 2,837 annotated regions were expressed *in vitro*, at 95.25% coverage, representing all broad functional gene categories in the genome. The expression profile also helped identify 518 potential operon structures involving 1,086 co-expressed pairs. We also identified 11 proteins with mutated/alternate start codons.

**Conclusions:**

The application of RNA-Seq based transcriptome profiling to structural gene annotation helped correct existing annotation errors and identify potential novel protein coding regions and sRNA. We used computational tools to predict regulatory elements such as promoters and terminators associated with the novel expressed regions for further characterization of these novel functional elements. Our study complements the existing structural annotation of *Mannheimia haemolytica *PHL213 based on experimental evidence. Given the role of sRNA in virulence gene regulation and stress response, potential novel sRNA described in this study can form the framework for future studies to determine the role of sRNA, if any, in *M. haemolytica *pathogenesis.

## Background

A systems-level understanding of organisms is not feasible by studying the functions of individual genes or proteins using reductionist approaches. It requires describing all molecular-level components that constitute building blocks of the system, identifying interactions among these components and determining regulatory modules to model emergent behavior [1]. As such, identifying all functional elements including genes, RNA, and proteins is a prerequisite to generating predictive models of biological response to biotic or abiotic perturbations. The genome sequence encodes all the necessary information required to decipher its functions. Therefore, genome sequencing, with concomitant structural annotation, i.e., identification of the functional elements within the genome, including genes, gene structures, open reading frames and regulatory motifs, is a critical step for conducting systems biology research. It is imperative that current and up-to-date knowledge of molecular level components exists for a genome sequence. Therefore, re-annotation is key to identifying these fundamental components of biological processes.

*De novo *assembly of a genome is followed by mapping of functional elements using computational methods. Computational methods for prokaryotic gene annotation such as Gene Locator and Interpolated Markov ModelER (GLIMMER) [2] and GeneMark.hmm [3] use hidden Markov models [4] based on a sequence similarity measure generated from previously annotated genomes. These algorithms do not accurately identify all genes in the genome and sometimes result in errors, especially in positioning of translational start codons [5] and in the identification of small protein coding genes. Another major problem with computational approaches is over-annotation, which arises from the failure to discriminate between random open reading frames and those that are translated. Computational prediction of small non-coding RNA (sRNA), which lack sequence conservation in closely related species, has limited accuracy since transcriptional signal prediction (promoter and rho-independent terminator prediction) is also not accurate. Therefore, sRNA that regulate many biological processes, including virulence in bacterial pathogens, cannot be identified by computational approaches alone.

Experimental identification of expressed regions in the genome can help overcome some of the drawbacks of computational methods and is a complementary approach to computational genome annotation methods. DNA microarrays, serial analysis of gene expression (SAGE) or high throughput transcriptome sequencing technologies such as RNA-Seq, can all be used to measure genome expression [6-9]. Of these methods, RNA-Seq, which generates a single nucleotide resolution map of the transcriptome, can help annotate mRNA, non-coding RNA and sRNA, transcriptional structure of genes, and post-transcriptional modifications induced by alternate splicing in eukaryotes [10-13].

In this study, we report re-annotation of *M. haemolytica*, a gram-negative bacterial pathogen and one of the causative agents of bovine respiratory disease (BRD) in cattle. BRD is responsible for over $3 billion in losses to the cattle industry every year [14]. *M. haemolytica *is most commonly isolated in field cases of BRD [15] and is considered to be the primary pathogen for this disease. Due to its importance for disease etiology, the genome of a bovine strain of *M. haemolytica *was sequenced in 2006. However, to date, the 2.6 Mb *M. haemolytica *PHL213 genome sequenced with an 8.4× coverage, is still in its draft phase. Despite being sequenced 6 years ago, the *M. haemolytica *genome sequence has not seen any improvement in its quality. Therefore, we chose to conduct RNA-Seq based re-annotation of *M.haemolytica*. The single nucleotide resolution map generated helped identify novel protein coding regions, sRNA, correct annotation errors, and operon structures.

## Materials and methods

### RNA isolation

*M. haemolytica *PHL213 was cultured in brain heart infusion (BHI) to mid-log phase (OD_620 _= 0.8). Cells from a single culture were treated with RNAprotect reagent (Qiagen, Valencia, CA) and stored at -80°C for subsequent RNA isolation. Total RNA from this single culture was extracted using the RNeasy mini kit (Qiagen, Valencia, CA), following manufacturer's protocols. It is to be noted that this kit allows for the extraction of transcripts that are at least 200 nucleotides and larger. RNA preparations were treated with RNase-free DNAse (Invitrogen, Carlsbad, CA) and the integrity of the RNA was determined using Bioanalyzer 2100 (Agilent Technologies, Santa Clara, CA). RNA sample with RNA Integrity Number (RIN) of 8 was used for the RNA-Seq experiment. From total RNA, mRNA was enriched by removing the rRNAs using MICROBExpress™ kit (Ambion, Austin, TX). This enrichment step specifically removes large rRNAs; small RNAs (i.e., tRNA and 5S rRNA) are not removed. In the first step of the MICROBExpress™ kit procedure, total RNA was mixed with an optimized set of capture oligonucleotides that bind to the bacterial 16S and 23S rRNAs. Next, the rRNA hybrids were removed from the solution using derivatized magnetic microbeads. The mRNA remained in the supernatant and was recovered by ethanol precipitation and quantified by Bioanalyzer 2100. Our RNA preparation did not include entities < 200 nucleotides in length.

### RNA-Seq

A cDNA library was constructed using the Illumina mRNA-Seq sample prep kit (Illumina, San Diego, CA) with 100 ng RNA enriched for mRNA isolated from a single *in vitro *culture, following manufacturer's instructions. mRNA was chemically fragmented and randomly primed for reverse transcription and second-strand synthesis. The resulting cDNA was end-repaired and 'A' overhangs were added. Illumina paired-end sequence adaptors were ligated to the cDNA fragments. Fragments of approximately 200 bp were isolated from a 2% agarose gel and amplified (18 cycles) according to the Illumina protocol. Bioanalyzer 2100 (Agilent) was used to quantify and confirm the fragment size of each library. 1 nM of mRNA-seq library sample prepared for sequencing on the Illumina GAII (San Diego, CA) was denatured and diluted to 6 pM for clustering (v2) according to the manufacturer's protocol. Single read sequencing of the clustered flow cell was performed using Illumina's SBS chemistry (v3) and SCS data analysis pipeline v2.4. Flow-cell image analysis and cluster intensity calculations were carried out by Illumina Real Time Analysis (RTA v1.4.15.0) software. Subsequent base-calling was performed using the Illumina GA Pipeline v1.5.1 software. The resulting Illumina reads were quality-filtered by removing reads containing Ns.

### Alignment

The sequencing experiment produced 9,055,826 reads. FASTQ reads generated by Illumina were converted to Sanger FASTQ format using Perl scripts from the Mapping and Assembly with Qualities (MAQ) software package [16]. Reads (Sanger FASTQ format) were mapped to the 2.6 Mb *M. haemolytica *PHL213 [GenBank: AASA00000000] reference genome using Bowtie [17]. The parameters in Bowtie that control the speed and sensitivity were adjusted as follows: reads with no more than 2 mismatches per read (n = 2) were aligned, and any reads mapped to more than one location across the genome (ambiguous reads) were discarded (m = 1). Post alignment, a human-readable sequence alignment/map (.SAM) format file was converted to a "pileup" format file using SAMTools [18]. This pileup file contains the count of reads per base aligned to each location across the length of the genome. The SAM file was also converted into a binary alignment/map (.BAM) format. These BAM formatted files are necessary for visualization of read alignments in Artemis viewer. The Artemis browser enabled the visual/manual inspection of alignment results in the context of the existing genome annotation. The pileup file, in conjunction with the annotation information of *M. haemolytica *PHL213, was processed using in-house Perl [19] scripts. Data generated from the RNA-Seq experiment was submitted to the NCBI Sequence Read Archive [SRA049621.1] as reads in FASTQ format [SRR402063.1] and the .BAM alignment file [SRR402079.4] generated by aligning the reads to the reference genome.

### Analysis of expressed intergenic regions

Identifying expressed regions within the genome that have not been previously annotated will improve the existing structural annotation of the *M. haemolytica *PHL213. Prior to the analysis of expressed regions in the genome, we determined the signal to noise ratio cutoff for background expression using the pileup file. Coverage depth (reads per base) greater than the lower tenth percentile of all reads was considered to be expressed and in this dataset, this corresponded to 7 reads/base [8,20]. Based on this read/base cutoff, expressed intergenic regions (EIRs) were identified by applying an additional length cutoff of 70 bp. Shorter regions (less than 70 bp) were discarded to reduce the number of false positives. Custom Perl scripts were written to parse the pileup file and the existing genome structural annotation to identify (i) expressed annotated regions, (ii) expressed regions previously not annotated and, (iii) regions that are annotated but are not expressed. All EIRs were further analyzed using BLASTX [21] searches to determine their protein coding potential. If an EIR was found to be a perfect match (~100% coverage) for a protein, it was classified as a putative novel protein coding region. All EIRs with partial BLASTX hits were evaluated for the presence of an alternate start site or mutation in the start or stop codon associated with the annotated region. If the BLASTX search revealed a frameshift mutation, the EIR and the gene associated with the frameshift mutation were classified as a frameshift. EIRs with poor BLASTX hits and without any association to genes containing annotation errors were excluded from further analysis. EIRs without BLASTX hits were considered to be potential small non-coding RNA.

The Prokaryotic Promoter Prediction (PPP) program [22] (from PePPER suite [23]) and Transterm HP [24] were used to predict promoters and rho-independent terminators, respectively, in the forward and reverse strands of the *M. haemolytica *PHL213 genome. The locations of promoters and terminators were organized into .GFF files. A Perl script was written to identify putative sRNA i.e. EIRs with promoters or terminators associated to their loci. EIRs with no computationally-predicted promoters or rho-independent terminators were searched against the Rfam database [25] to determine whether these sequences were annotated in Rfam. EIRs that could not be classified as sRNA by Rfam were excluded from further analysis.

### Analysis of expressed annotated regions

Using the annotation information (gene loci) of *M. haemolytica *PHL213 and the pileup file, all annotated regions that were expressed above the background signal to noise ratio cutoff with at least 60% coverage were considered to be expressed, which accounts for uniform evaluation of varying gene lengths. Similar measures have been used in other transcriptome profiling studies [8,26,27]. Annotated regions below 60% coverage were considered as 'not expressed' under the current experimental conditions. After having identified expressed genes, operon structures within the genome were also defined. The first step towards identifying an operon was to identify co-expressed pairs of coding regions. Two regions were considered to be co-expressed when they were identified as expressed on the same strand (5' to 3' or 3' to 5') and the region between them was also expressed. After such co-expressed pairs were identified, they were extended to construct operons by including additional co-expressed pairs in the vicinity satisfying the same conditions for co-expression as described earlier. Operon structures identified by RNA-Seq were compared to the computationally-predicted operon structures described by the Database for prOkaryotic OpeRons (DOOR) [28] for cross validation.

## Results

### Read alignment to the *M. haemolytica *PHL213 genome

The *M. haemolytica *PHL213 is a 2.6 Mb draft genome containing 2,837 annotated regions of which 2,695 are protein coding with a 40% G+C content [29]. For structural annotation of *M. haemolytica *at the RNA level, the transcriptome of *M. haemolytica *PHL213 was sequenced using RNA-Seq. Sequencing-based analysis of the transcriptome overcomes the limitations of the hybridization-based microarray approach. Head-on comparison of RNA-Seq with microarrays has shown that RNA-Seq has negligible technical variability [30], making it possible to obtain a reliable estimate of gene expression without replicate analysis. Therefore, we applied RNA-Seq for re-annotation of *M. haemolytica *and conducted the analysis from a single *in vitro *experiment. Reads with an average length of 76 bp generated on the Illumina platform were mapped onto the reference genome using the Bowtie read alignment program. Bowtie is an ultrafast, memory efficient alignment program that uses the Burrows-Wheeler transform [31] with a novel quality backtracking algorithm that permits mismatches. Bowtie performs better than Short Oligonucleotide Analysis Package (SOAP) [32] and MAQ, and its sensitivity at aligning reads is as good as both SOAP and MAQ. Of the 9,055,826 reads generated by Illumina, 3,917,458 reads (43.26%) that mapped uniquely to the genome were used for downstream analysis. 2,989,603 reads (33.01%) failed to align due to mismatches. The remaining 2,148,765 reads (23.73%), which mapped to more than one location in the genome (ambiguous reads), were excluded from analysis. For annotation purposes, reads that map to unique locations alone are used [8,33-37]. The cutoff value for true-positive expression of a coding region of 7 reads/base was calculated from the expression (number of reads per base) in the tenth percentile of all reads [8,20], as we did earlier for RNA-Seq based re-annotation of another BRD pathogen *Histophilus somni *[8].

### Expressed intergenic regions

**Figure 1:**
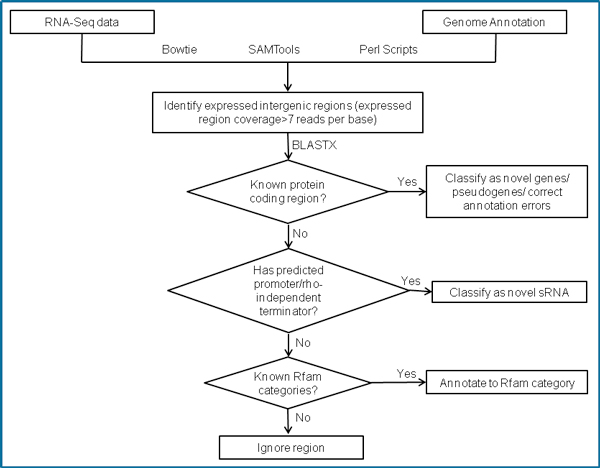
**Data analysis flow-chart**. RNA-Seq reads are aligned to the genome using Bowtie to generate a single nucleotide resolution map. Mapped reads analyzed in the context of existing annotation using SAMTools and Perl scripts generate expressed intergenic regions (EIRs). Homologs for EIRs, if present, are identified by BLASTX and are classified accordingly. An EIR with no BLASTX matches is subjected to computational search for regulatory elements (promoter/rho-independent terminator) in its vicinity. An EIR with a regulatory signal is classified as potential sRNA. EIR without BLASTX matches and predicted transcriptional signals are searched against Rfam database.

We used the existing annotation of open reading frames in *M.haemolytica *PHL213 i.e., locus of each gene in the genome and reads identified as expressed by RNA-Seq, to identify expressed intergenic regions (EIRs). We identified 630 EIRs, previously un-annotated as expressed, of a minimum length of 70 bp. Each EIR was further characterized by adding computationally-predicted promoter and rho-independent terminators. Prokaryotic Promoter Prediction (PPP) identified 11,847 promoter regions and Transterm HP identified 1,204 rho-independent terminator regions, in forward and reverse strands of the genome. Identified EIRs, in conjunction with existing gene annotation information and loci of regulatory signals, were subjected to the analysis workflow described in Figure 1.

**Figure 2:**
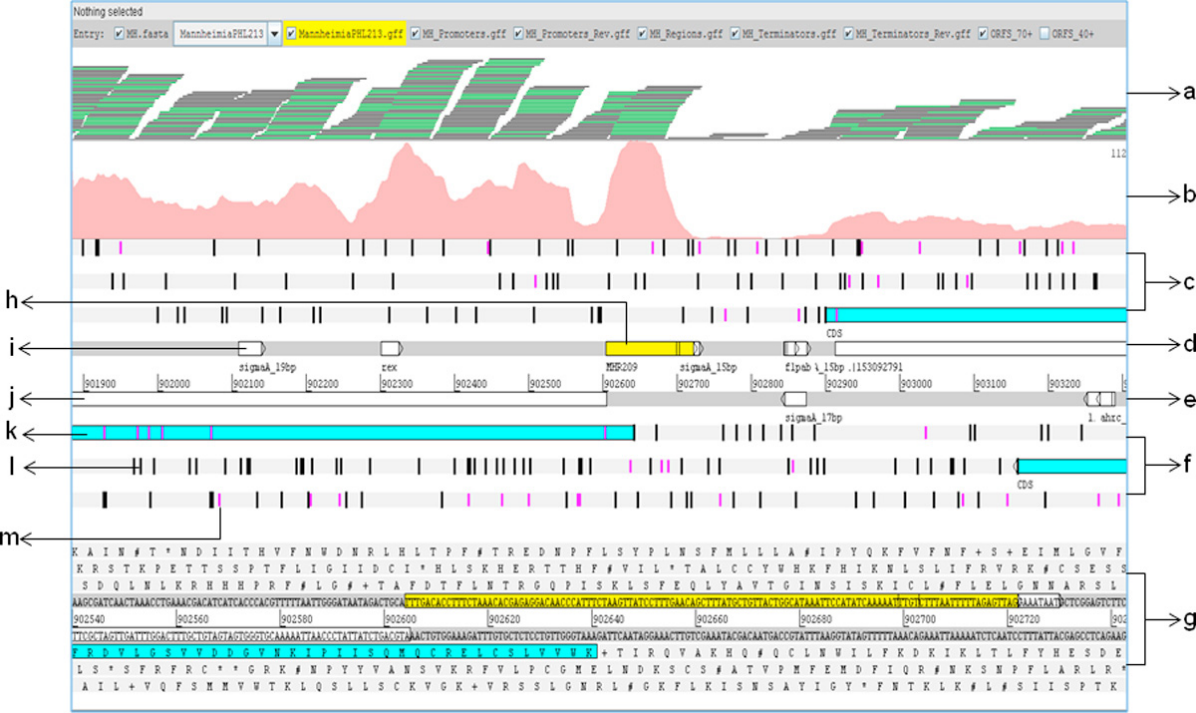
**Overview of Artemis genome browser**. Description of the tracks used for data analysis in Artemis genome browser. (a) reads aligned to the genome (b) expression portrayed as coverage graph (c) reading frames 5'-3', (d) forward strand of the genome, (e) reverse strand of the genome, (f) reading frames 3'-5', (g) amino acid sequences corresponding to the 6 reading frames, (h) expressed intergenic region, (i) computationally predicted regulatory signal (promoter/rho-independent terminator), (j) annotated gene (white), based on *M. haemolytica *Genbank accession # AASA00000000, (k) ORFs (blue) of a specified minimum length, predicted by Artemis between two consecutive stop codons, (l) stop codons in all six reading frames (black) and, (m) start codons in all six reading frames (pink).

Artemis is a genome browser and annotation tool that allows visualization of sequence features, next generation sequencing data, and the results of the analyses within the context of the genome sequence [38]. The Artemis genome browser illustrates all the six reading frames of the genome sequence along with the translated amino acid sequences, start and stop codons, as well open readings frames across the length of genome (Figure 2). We visualized the alignment file generated by Bowtie, the gene annotation file, promoter and terminator loci, and EIRs in Artemis. Artemis generated a base coverage graph, giving a pictorial representation of the expression in various regions of the genome.

### Novel protein coding regions

**Figure 3:**
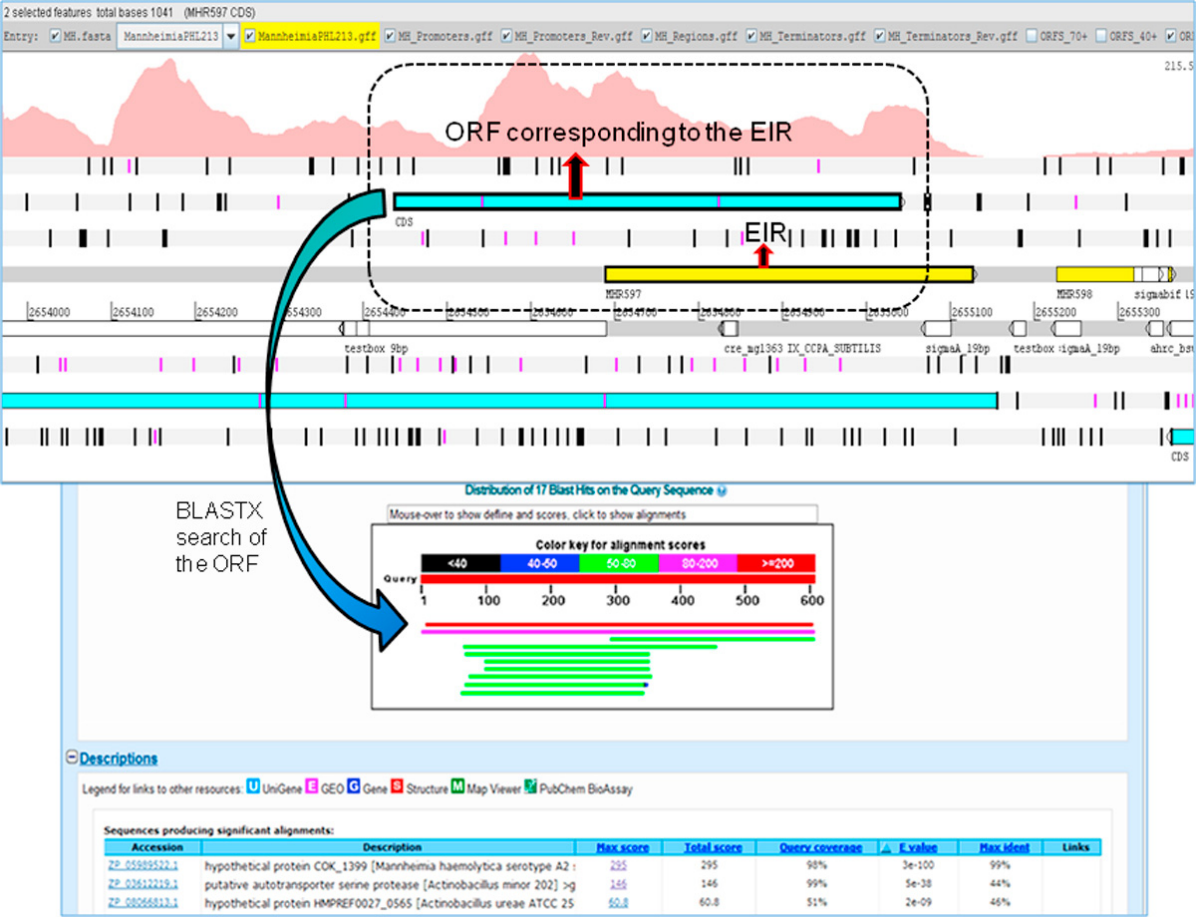
**A novel protein coding region**. Identification of novel protein coding regions using expressed intergenic region (EIR, yellow) and its corresponding open reading frame (ORF, blue). A BLASTX search of the ORF identified a full length match to a protein with 98% coverage. The coverage graph for the region of interest is shown (dotted line).

The protein coding potential of EIRs was determined by conducting BLASTX searches with the translated nucleotide sequence of EIRs, against the protein database containing all bacterial species. BLASTX results showed that 14 EIRs had full length matches to target sequences, indicating their potential for coding proteins. The Artemis browser was used to identify the boundaries of these 14 potential novel protein coding regions (Figure 3). These novel protein coding regions had an average G+C content of approximately 46%. The length of these regions was between 37 to 200 amino acids. While the RNA-Seq experiment itself was not strand specific, strand specificity of novel protein coding regions was inferred from the proteins identified as ~100% matches to these EIRs in BLASTX. EIR MHP4 aligned to PG1 protein of *Lactobacillus crispatus *ST1 while MHP12 aligned to serine acetyltransferase of *Haemophilus influenzae *NT127. The rest of the EIRs (Table 1) aligned to proteins classified as hypothetical.

**Table 1:** Potential novel protein coding regions identified in *M. haemolytica *PHL213

Protein ID	Protein Start	Protein End	Length	Strand	Protein description	Organism
MHP1	5578	5898	107	-	hypothetical protein HPS_04442	*Haemophilus parasuis *29755
MHP2	532998	533216	73	-	conserved hypothetical protein	*Methylococcus capsulatus *str. Bath
MHP3	608419	608553	45	-	hypothetical protein COI_2717	*M. haemolytica *serotype A2 str. OVINE
MHP4	740406	740837	144	-	PG1 protein	*Lactobacillus crispatus *ST1
MHP5	740634	740759	42	+	hypothetical protein GG9_1745	*Haemophilus haemolyticus *M19501
MHP6	740709	740936	76	-	hypothetical protein Csp_D29610	*Curvibacter *putative symbiont of Hydra magnipapillata
MHP7	901072	901212	47	+	hypothetical protein COK_2315	*M. haemolytica *serotype A2 str. Bovine
MHP8	1168344	1168478	45	-	hypothetical protein COI_2717	*M. haemolytica *serotype A2 str. Ovine
MHP9	1256181	1256366	62	+	hypothetical protein COI_1129	*M. haemolytica *serotype A2 str. Ovine
MHP10	1460962	1461081	40	-	hypothetical protein COK_1081	*M. haemolytica *serotype A2 str. Bovine
MHP11	2531614	2531724	37	-	hypothetical protein COK_2196	*M. haemolytica *serotype A2 str. Bovine
MHP12	2605237	2605434	66	-	serine acetyltransferase	*Haemophilus influenzae *NT127
MHP13	2639084	2639221	46	-	hypothetical protein COK_0003	*M. haemolytica *serotype A2 str. Bovine
MHP14	2654438	2655037	200	+	hypothetical protein COK_1399	*M. haemolytica *serotype A2 str. Bovine

Potential protein loci, length (aa), inferred strand direction, description of the corresponding BLASTX protein match.

### Corrections made to the existing genome annotation

**Figure 4:**
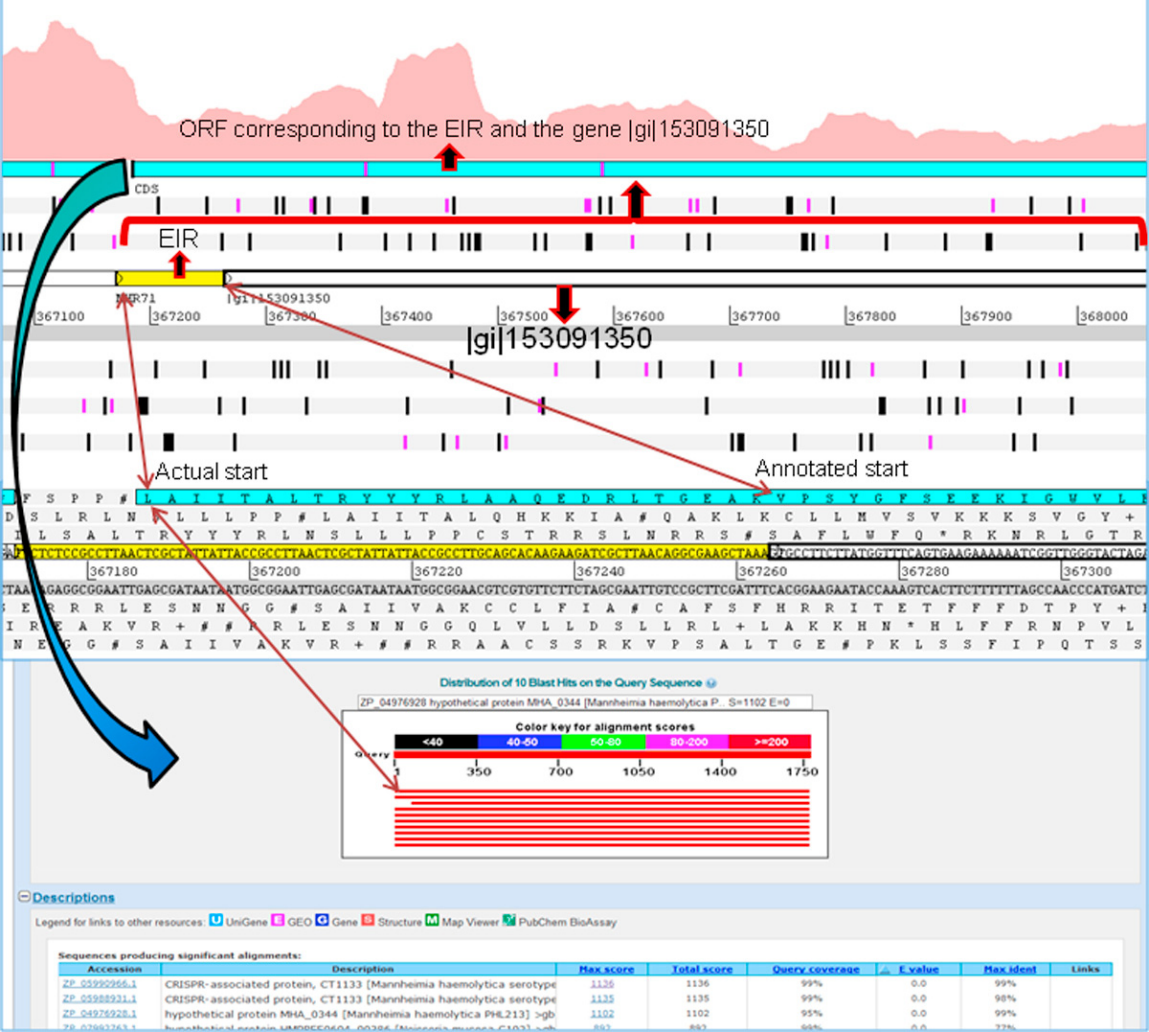
**Mutated start site**. Visualization of a gene identified with a mutation in the start site in Artemis genome browser. RNA-Seq based coverage graph clearly shows expression upstream of the annotated start site of the gene |gi|153091350. When the ORF encompassing the gene |gi|153091350 and the expressed intergenic region (EIR, yellow) upstream of the gene, was used for conducting BLASTX searches, we identified a full length match suggesting that the actual start site is at 'leucine' (probably mutated), instead of 'valine' (based on existing annotation).

Artemis genome browser creates open reading frames (ORFs) of a desired minimum length. It identifies ORFs as regions between two consecutive stop codons with the specified minimum length. Thus ORFs corresponding to EIRs can be generated and visualized in this browser. RNA-Seq based expression in relation to the existing genome annotation, when visualized in Artemis, enabled the identification of the actual locus and length for some of the annotated proteins. We identified 4 genes with a mutated start codon. This anomaly could be the result of computational gene prediction programs identifying the next available "AUG" as the start codon (Figure 4). Our observation is substantiated by the consecutive expression of an identified EIR preceding the 5' region of these genes. 4 genes had a mutation that led to the replacement of start codon by a leucine (L).

**Figure 5:**
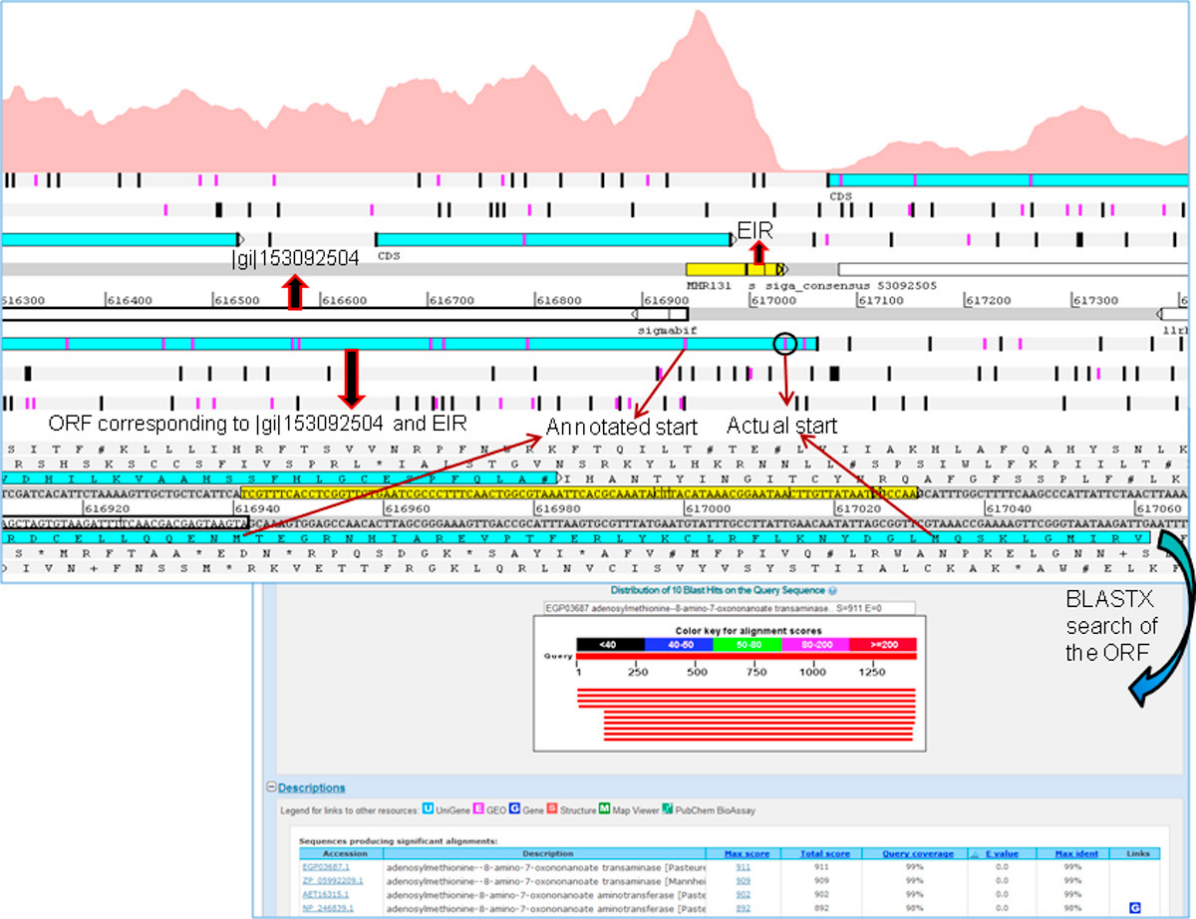
**Alternate start site**. Artemis genome browser identified an alternate start site for the gene |gi|153092504. The coverage graph for this gene showed that the start codon based on expression is different from annotated start site. In the 5' upstream region of annotated start site, there are two methionines that could be potential start sites. BLASTX search identified a full length match, and confirmed one on these two methionines as the actual start site.

**Figure 6:**
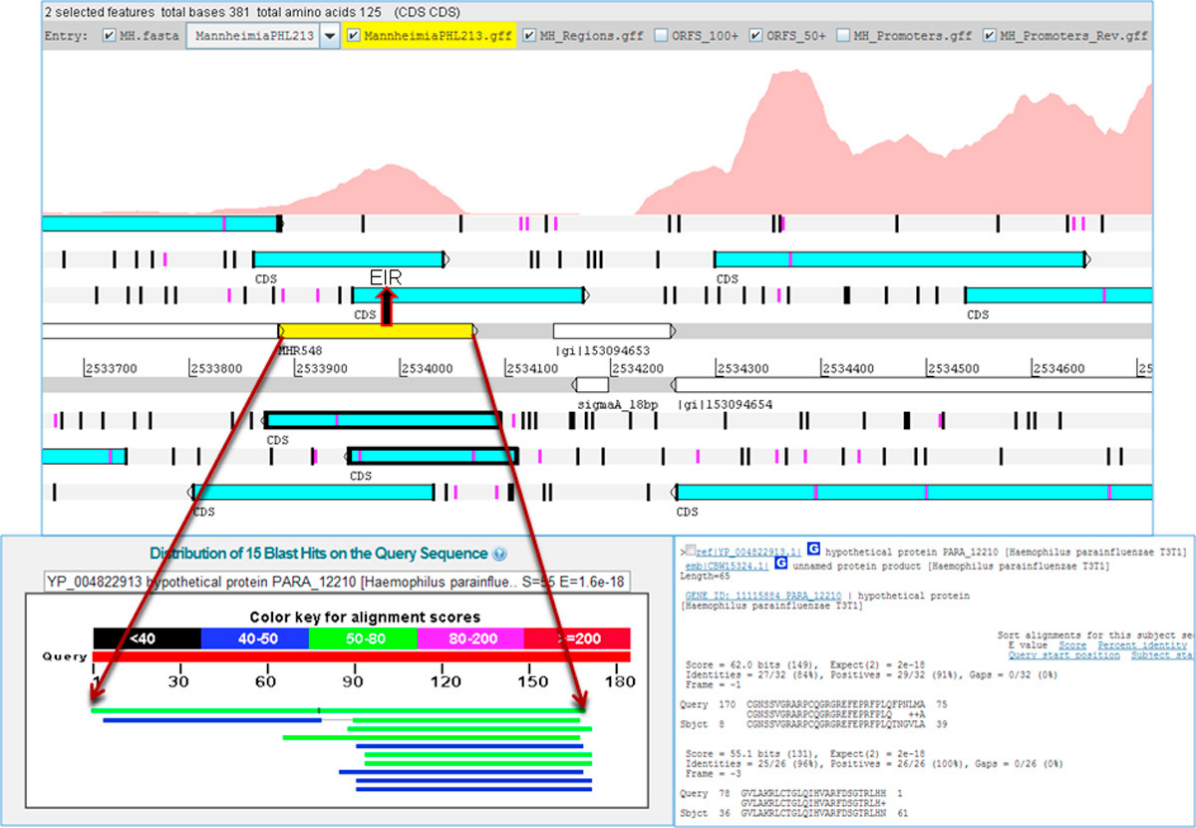
**Frameshift mutation**. An expressed intergenic region (EIR, yellow) and its corresponding ORFs (highlighted) visualized in Artemis genome browser indicate a possible frameshift mutation. BLASTX search of the EIR identified a full length match, confirming the frameshift mutation.

Since Artemis allows marking the start codons within an ORF, it is possible to identify alternate start sites, if any, for any ORF associated with an EIR. ORFs created from EIRs in Artemis revealed possible alternate starts sites for 7 genes. BLASTX searches of the EIR and its translated protein revealed that the actual start site varied with respect to previous annotation (Figure 5). Where there was a discrepancy between the existing annotation and the current transcriptome based identification of start site, the consensus of start site of similar proteins identified in BLASTX was used to determine the actual start site. The suggested revisions to existing annotation are in Table 2 (detailed results in Additional file 1). BLASTX searches of EIRs also revealed mutations that lead to disruption of a protein coding region, resulting in a frameshift (Figure 6). Two such EIRs which had BLASTX alignments revealed frameshifts which would otherwise be protein coding regions (Additional file 1).

**Table 2:** Suggested corrections made to the existing annotation of *M. haemolytica *PHL213

Protein ID	Gene start	Gene end	Gene length	Protein length	Strand	Corrected start	Corrected end	Corrected gene length	Corrected protein length	Correction Type
MHA_0097	91433	92209	777	258	-	91433	92281	849	282	Mutated Start (L)
MHA_0304	314414	316213	1800	599	+	314210	316213	2004	667	Mutated Start (L)
MHA_0344	367264	368961	1698	565	+	367186	368961	1776	591	Mutated Start (L)
MHA_0410	430731	431060	330	109	+	430650	431060	411	136	Alternate Start
MHA_0591	615640	616941	1302	433	-	615640	617052	1413	470	Alternate Start
MHA_0736	750172	751350	1179	392	+	750121	751350	1230	409	Alternate Start
MHA_0819	821700	822038	339	112	+	821472	822038	567	188	Mutated Start (L)
MHA_1225	1209899	1211335	1437	478	+	1209791	1211335	1545	514	Alternate Start
MHA_1712	1697137	1698519	1383	460	+	1696987	1698519	1533	510	Alternate Start
MHA_2666	2591266	2592276	1011	336	-	2591266	2592360	1095	364	Alternate Start
MHA_2723	2659955	2660545	591	196	-	2659955	2660692	738	245	Alternate Start

**P**reviously annotated gene locus, length and strand specificity. Observed locus based on RNA-Seq expression along with gene length (bp) and protein length (aa) and the description of the annotation error. In case of a mutated start, the amino acid start codon identified by RNA-Seq.

### Small RNA

**Figure 7:**
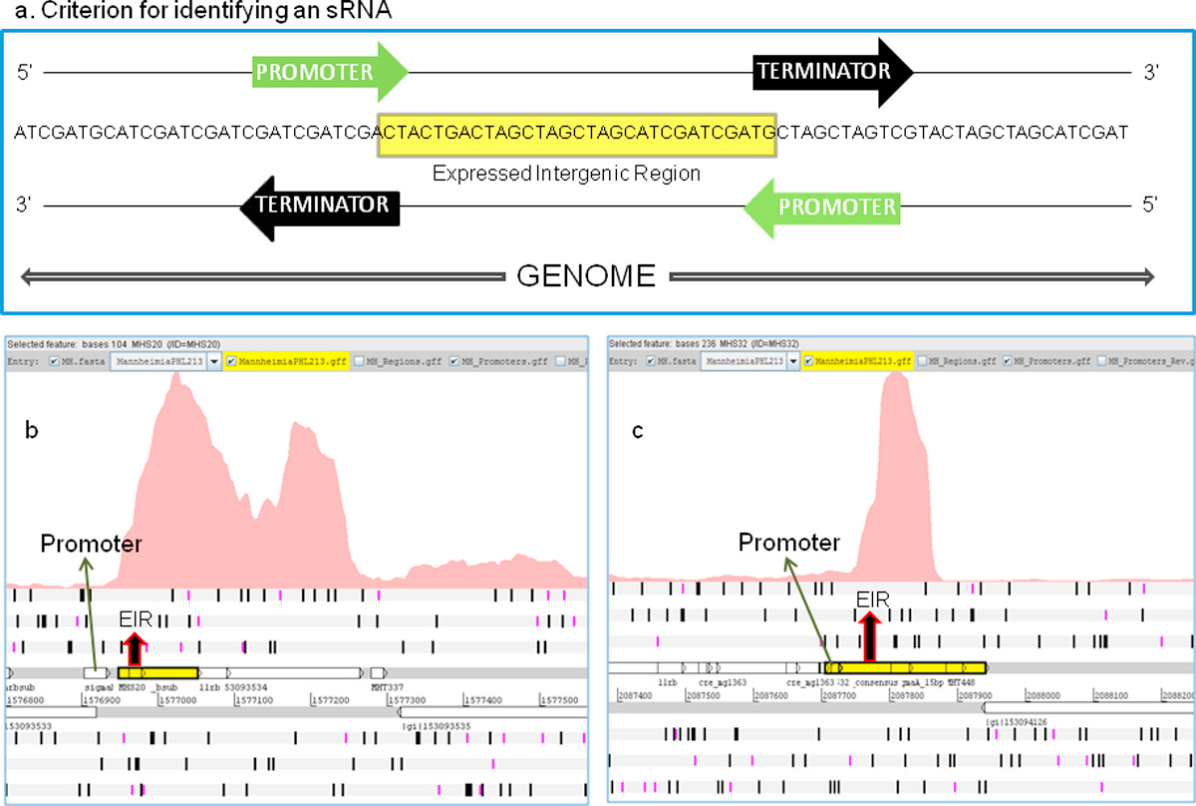
**Identification of potential sRNA**. (a) Criterion for identifying potential sRNA. If a promoter located upstream of the expressed intergenic region (EIR) or a rho-independent terminator located downstream of the EIR, either in the forward or the reverse strands of the genome is identified, then the EIR is classified as a potential sRNA. (b) & (c) a promoter was identified upstream of the EIR in both the cases and the EIRs were classified as sRNA.

Small RNA are known to have regulatory roles in *Escherichia coli, Staphylococcus aureus, Pseudomonas aeruginosa, Vibrio cholera *and many other bacterial pathogens [39]. Genome-scale identification of sRNA using RNA-Seq is reported for *E. coli *[40] and *Vibrio cholerae *[41], among other pathogenic bacteria. The identification of the loci of sRNA in the genome is an important pre-requisite for understanding their role in modulating bacterial physiology and virulence [42]. sRNA are synthesized by RNA polymerase (RNAP) in a manner analogous to the synthesis of any RNA in bacteria (mRNA, rRNA, tRNA); sRNA promoters could be regulated by transcription factors or use of alternative sigma factors [43]. Therefore, the presence of promoters and terminators for potential sRNA [8,44] identified by experimental approaches like RNA-Seq, increases the confidence in their identification. Although the RNA extraction protocol used in this study does not facilitate extraction of smaller transcripts and strand specificity is lost during cDNA synthesis, we identified potential sRNA. EIRs with no protein coding potential, as observed via BLASTX searches, were considered to be candidate sRNA. It is possible that EIRs with no BLASTX matches are non-conserved ORFs; since there are no *in silico *methods to validate this assumption, we chose to consider all EIRs with no BLASTX as candidates for small RNA analysis. Candidate sRNA loci were searched for the presence of a promoter or terminator. For 44 EIRs that had no BLASTX matches, a promoter or a rho-independent terminator was identified either on the forward or the reverse strand (Figure 7) of their locus. Promoters/terminators were present in the transcriptional regulatory regions, i.e., a promoter was present in the -1 to -35 region or a terminator was present in the +1 to +20 position at the end of the EIR. Therefore, we classified the 44 candidate sRNA as potential novel small RNA in the *M. haemolytica *PHL213 genome (Table 3). The average length of the identified novel sRNA was approximately 100 bp and ranged between 70 to 253 bp. The average G+C content of sRNA was 34.35%, which is relatively lower than the G+C content of the genome. All identified sRNA had a promoter associated with their locus and sRNA MHS17 also had an associated terminator. When sequences of the identified sRNA were searched in the Rfam [25] database to identify their function, no matches were found.

**Table 3:** Putative novel sRNA identified in *M. haemolytica *PHL213

sRNA_ID	Start	End	Length(bp)	Flanking gene (left)	Flanking gene (right)	Promoter (Strand)	BLASTN match
MHS1	219874	219955	82	MHA_0231|-	MHA_0232|lamB	Y(+)	
MHS2	309458	309563	106	MHA_0300|-	MHA_0301|-	Y(+)	*Haemophilus parasuis *SH0165
MHS3	319131	319229	99	MHA_0307|era	MHA_0308|recO	Y(+)	*Mannheimia granulomatis *str. P1135/26
MHS4	353514	353599	86	MHA_0333|res	MHA_0334|gyrB	Y(+)	
MHS5	428735	428820	86	MHA_0405|-	MHA_0406|-	Y(+)	Bacteriophage phi-MhaA1-BAA410
MHS6	601909	601989	81	MHA_0568|-	MHA_0569|-	Y(+)	*Haemophilus parasuis *SH0165
MHS7	694168	694238	71	MHA_0694|uppS	MHA_0695|-	Y(+)	
MHS8	728194	728446	253	MHA_0724|-	MHA_0725|hemL	Y(+)	
MHS9	760853	760946	94	MHA_0748|dnaA	MHA_0749|ccmA	Y(+)	
MHS10	809903	810012	110	MHA_0806|-	MHA_0807|rluC	Y(+)	
MHS11	849372	849453	82	MHA_0843|pstC	MHA_0844|pstS	Y(+)	
MHS12	962811	962887	77	MHA_0957|-	MHA_0958|-	Y(+)	
MHS13	1004733	1004837	105	MHA_1000|crp	MHA_1001|murB	Y(+)	
MHS14	1006880	1006950	71	MHA_1003|-	MHA_1004|hxuA	Y(+)	
MHS15	1111723	1111820	98	MHA_1103|-	MHA_1104|-	Y(+)	
MHS16	1164081	1164188	108	MHA_1167|-	MHA_1168|-	Y(+)	*Histophilus somni *2336
MHS17	1422454	1422537	84	MHA_1444|-	MHA_1445|-	Y(+)	
MHS18	1427285	1427368	84	MHA_1449|-	MHA_1450|-	Y(+)	
MHS19	1544002	1544093	92	MHA_1562|-	MHA_1563|dapA	Y(+)	
MHS20	1576948	1577051	104	MHA_1597|-	MHA_1598|rpmE	Y(+)	
MHS21	1580406	1580478	73	MHA_1601|-	MHA_1602|lon	Y(+)	
MHS22	1590485	1590566	82	MHA_1610|-	MHA_1611|-	Y(+)	
MHS23	1674686	1674757	72	MHA_1690|leuA	MHA_1691|-	Y(+)	
MHS24	1705600	1705693	94	MHA_1719|-	MHA_1720|uspA	Y(+)	
MHS25	1784761	1784848	88	MHA_1796|-	MHA_1797|-	Y(+)	
MHS26	1842730	1842833	104	MHA_1864|rpoZ	MHA_1865|-	Y(+)	
MHS27	1861740	1861815	76	MHA_1884|-	MHA_1885|-	Y(+)	
MHS28	1872042	1872162	121	MHA_1892|-	MHA_1893|-	Y(+)	
MHS29	1902920	1903001	82	MHA_1926|aroE	MHA_1927|uvrD	Y(+,-)	
MHS30	1977320	1977411	92	MHA_2021|-	MHA_2022|-	Y(+)	
MHS31	2054477	2054562	86	MHA_2099|ansB	MHA_2100|-	Y(+)	
MHS32	2087705	2087940	236	MHA_2131|-	MHA_2132|-	Y(+)	
MHS33	2135304	2135406	103	MHA_2171|mtlD	MHA_2172|-	Y(+)	*Haemophilus ducreyi *strain 35000 HP
MHS34	2141830	2141953	124	MHA_2185|tolQ	MHA_2186|tolR	Y(+)	
MHS35	2233877	2234009	133	MHA_2261|hmbR2	MHA_2262|-	Y(+)	
MHS36	2245148	2245226	79	MHA_2272|-	MHA_2273|purM	Y(+)	
MHS37	2333531	2333628	98	MHA_2360|-	MHA_2361|gntK	Y(+)	
MHS38	2438972	2439053	82	MHA_2487|nagB	MHA_2488|nagA	Y(+)	
MHS39	2623678	2623787	110	MHA_2696|dinJ	MHA_2697|-	Y(+)	
MHS40	2624952	2625021	70	MHA_2698|miaA	MHA_2699|hfq	Y(+)	
MHS41	2646708	2646793	86	MHA_2712|-	MHA_2713|tpx	Y(+)	
MHS42	2691938	2692007	70	MHA_2762|ansB	MHA_2763|pyrG	Y(+,-)	
MHS43	2703602	2703676	75	MHA_2776|-	MHA_2777|trmU	Y(+)	*Actinobacillus pleuropneumoniae *serovar 3 str. JL03
MHS44	2755982	2756108	127	MHA_2824|mgsA	MHA_2825|thrC	Y(+)	

Identified potential sRNA, their locus, length (bp), flanking genes, predicted promoter and its strand specificity, top BLASTN hit (if any).

EIRs with no BLASTX matches, predicted promoter, or a rho-independent terminator, were searched against the Rfam database to identify potential matches with any of the known conserved RNA families in the database. Five EIRs mapped to five different functional categories within Rfam, shown in Table 4. MHS45 was classified as bacterial signal recognition particle RNA, a conserved ribonulceoprotein that directs movement of proteins within the cell and aids their secretion. MHS46 was classified as MOCO RNA motif which is presumed to be a riboswitch that binds to molybdenum cofactor or related tungsten cofactor. MHS47 was classified as a thiamine pyrophosphate (TPP) riboswitch that binds directly to thiamine pyrophosphate to regulate gene expression. MHS48 was classified as an alpha operon ribosome binding site that binds to ribosomal protein S4 which acts as a translational repressor. MHS49 was annotated by Rfam as a *gcvB *RNA that encodes small non-coding RNA involved in the regulation of amino acid transport systems and amino acid biosynthetic genes. All predicted functions will need to be validated by further experimentation.

**Table 4:** Putative sRNA identified in identified in *M. haemolytica *PHL213 using the Rfam database

sRNA_ID	Start	End	strand	rfam-id	rfam accession	G+C content
MHS45	698093	698190	-	Bacteria_small_SRP	RF00169	49
MHS46	902602	902713	-	MOCO_RNA_motif	RF01055	33
MHS47	2002337	2002433	+	TPP	RF00059	51
MHS48	2339489	2339566	-	Alpha_RBS	RF00140	32
MHS49	2687066	2687209	-	GcvB	RF00022	34

EIRs that do not have either a BLASTX hits or predicted promoter or rho-independent terminator, but with matches in Rfam database. sRNA locus, strand specificity and description based on Rfam.

### Gene expression and operons

The *M. haemolytica *PHL213 genome consists of 2,837 annotated genes, 2,695 of which code for proteins. Genes were considered to be expressed if 60% of the gene length had at least 7 reads aligned/nucleotide. Based on this criteria, 2,506 of all annotated regions in the genome (87.63%) were identified as expressed with 95.25% coverage i.e. approximately 95% of the sequence of the annotated region had at least 7 reads aligned/nucleotide. Expressed annotated genes and their coverage are documented in Additional file 2.

Functional analysis of the expressed annotated regions was based on the existing annotation of *M. haemolytica *genome available at NCBI. It is interesting to note that genes that are described as virulence factors are also expressed under normal culture conditions. For example, genes related to leukotoxin (MHA_0253, MHA_0254, MHA_0255 and MHA_0266), an important virulence factor, were all expressed. Also, the 12 capsule forming genes whose role in virulence includes adherence to host and resistance to serum-mediated killing and phagocytosis [45,46] were all found to be expressed. In addition to these, we also found that 40 genes associated with lipopolysaccharide or lipoproteins and contribute to virulence by initiating an inflammatory cytokine response [45,47] to be expressed. Genes responsible for forming the type IV pilus associated with *M. haemolytica *that is responsible for DNA uptake, adhesion, and motility [48] were expressed. Filamentous hemagglutinin genes of *M. haemolytica *(MHA_0866, MHA_0867), responsible for adhesion to host mucosa [49], were expressed. Adhesins play an important role in virulence, and all annotated genes related to this function, such as MHA_2262, MHA_0708, MHA_2492, MHA_2701, MHA_1367, MHA_0563 and MHA_2800, among others, were all identified as expressed in our experiment. Genes responsible for resistance towards antibiotics such as β-lactams, tetracycline, streptomycin, and sulfonamides [45] in *M. haemolytica *were also expressed. Annotated regions that were not expressed had coverage of only 30%. Of the 331 annotated regions that were not expressed 236 were annotated as "hypothetical proteins" and 26 were "hypothetical bacteriophage proteins."

Using expression patterns of coding regions, we identified paired gene expression and operon structures. RNA-Seq based operon structures were compared to the computationally predicted structures using DOOR [28]. We identified 1,086 co-expressed pairs of genes that could be organized into 518 potential operons. DOOR predicted 1,295 co-expressed pairs forming 599 operons (Additional file 3). The overlap between RNA-Seq based and DOOR-based co-expressed pairs was 854. Our study identified relatively fewer co-expressed pairs as compared to DOOR. This could be due to the fact that 331 of the 2,837 annotated regions were not expressed in our dataset. Furthermore, this method cannot detect genes whose expression is suppressed by polar mutations. The single nucleotide resolution map enabled the identification of co-expressed pairs and definition of operon structures and regulatory patterns. Availability of operon structures will facilitate understanding the coordinated regulation of genes in *M. haemolytica *to moderate metabolic pathways under different environmental conditions.

## Discussion

Identification of all functional elements of the genome is fundamental to understanding the dynamics of biological processes that occur within any living organism. Gene models are available for sequenced genomes that are based on computational approaches. However, a number of recent studies highlight the need for genome re-annotation, prior to conducting holistic systems biology analyses. Experimental approaches, at times, shed light on regions of the genome where computational methods of structural annotation fail. Re-annotation studies of several species including disease causing pathogens have revealed numerous genes, regulatory regions and complex metabolic pathways that remained undetected based on the initial annotation [8,50-55]. In this study, we applied a combinatorial approach i.e. RNA-Seq based transcriptome analysis in conjunction with computational resources, to structurally annotate a bacterial genome at the RNA level. For the first time, we report RNA-Seq based annotation of the genome of *M. haemolytica *PHL213, one of the primary pathogens of Bovine Respiratory Disease in cattle [56]. Its genome was sequenced with 8.4× coverage, and is in draft phase since 2006.

We have recently re-annotated *Histophilus somni *2336, another BRD pathogen belonging to *Pasteurellaceae *like *M. haemolytica*. RNA-Seq based transcriptome analysis identified 38 novel protein coding regions and 82 sRNA in *H. somni *[8]. Compared to the draft genome for *M. haemolytica, H. somni *has a complete genome sequence. Yet, re-annotation of this genome identified a number of functional elements missed in the initial annotation. The relatively poor quality of the existing structural annotation of *M. haemolytica *can be enhanced by re-annotation, and this was the motivation behind the current study.

Re-annotation enabled us to fix errors in existing annotation. A mutation that might have occurred during replication could alter the structure of the gene in its vicinity. Computational methods, when predicting a gene, seek to identify an ORF and its putative start and stop codons to define gene boundaries. Mutations in the sequence between the start or stop codon of a gene might not actually affect gene prediction or may sometimes result in a frameshift. If the mutation is to occur in the start or stop codon itself, algorithms would seek to identify the next available start or stop codon. This would lead to alteration in gene locus and a subsequent gene annotation error. Such annotation errors cannot be detected without experimental validations. The single nucleotide resolution transcription map generated by RNA-Seq is one of the most efficient ways to detect such annotation errors. As described in our workflow (Figure 1), once EIRs overlapping a certain gene were identified, BLASTX searches of these regions helped in defining the actual boundaries and correct annotation errors, if any. Mutations leading to a frameshift can result in a gene being completely disrupted. Such frameshifts remain undetected by automated approaches, but can be identified by experimental approaches such as RNA-Seq used in this study. Genome-wide studies using experimental methods can help validate these predictions and improve the quality of annotation across genomes and eliminate errors from being transferred from one genome to another during annotation of novel assemblies.

Understanding coordinated regulation of gene expression in bacteria requires the description of operon structures in the genome. Prior to this study, operon structures were unavailable for *M. haemolytica*. Since computationally-predicted operon structures were unavailable, we first generated a set of computationally-predicted operons using DOOR (Additional file 3). RNA-Seq enabled us to identify expressed gene pairs that could be expanded into potential operons. Comparison of DOOR predicted operons with RNA-Seq based operons in *M. haemolytica *showed a major overlap and cross-validated the findings in both approaches. Thus re-annotation helped validate 599 operons predicted by DOOR. We also identified 233 co-expressed pairs that were not identified by DOOR. Since the strand specificity of expression is lost in RNA-Seq experiment described here, at best the operons identified in this study should be considered 'potential operons' that will require experimental validation in future studies. Furthermore, this experiment-based identification of co-expression will not be able to identify genes that are expressed in a polar fashion within the operon. Analysis of the functions of genes identified as expressed by RNA-Seq resulted in an interesting finding. Genes that are annotated as being virulence factors were identified as expressed under normal culture conditions. These results are consistent with our findings in *H. somni*. Our results indicate that the expectation of 'virulence factor' being expressed only during pathogenesis may not be accurate. It is possible that there is a basal pervasive level of expression of these factors and that it is the difference in the expression level that actually corresponds to virulence.

Computational methods for identification of sRNA are not accurate, and transcriptome profiling using deep sequencing methods can help identify novel sRNA. sRNA play a crucial role in adaptive response to stress by directly or indirectly regulating virulence genes [39], as shown in *Staphylococcus aureus *[57], *Pseudomonas aeruginosa *[58] and *Vibrio cholerae *[59,60]. However, a comprehensive understanding of sRNA regulatory roles during adaptive responses and pathogenesis is only possible after their identification. Despite the drawbacks in sample preparation and lack of strand specificity, we identified 44 potential novel sRNA. The identified novel sRNA were searched for homology in the sRNA database (sRNAdb) against other bacterial sRNA identified through similar transcriptomics studies and/or computational approaches [61]. Only 15 sRNA had partial alignments of 20-30 nucleotides and the remaining had very poor sequence conservation across the database (Additional file 4). We also compared the 44 sRNA identified in the *M. haemolytica *genome with 82 *H. somni *sRNA using 'BLAST 2 sequences' megablast [21]. No similarity was found, indicating poor consensus among non-coding RNA. These results suggest that regulation of sRNA is probably as diverse and as complex as gene or protein regulation.

The inherent limitations of our experimental setup i.e. lack of enrichment specifically for sRNA, lack of strand specificity information and lack of biological replicates, isolation of RNA at different stages of *in vitro *growth, etc, did not allow comprehensive identification of sRNA. Due to the same limitations, the identified gene co-expression also needs further validation work in future. However, as the results indicate, application of RNA-Seq enhanced the existing annotation of *M. haemolytica*. RNA-Seq based annotation is not the 'final' and conclusive step in identifying functional elements in this important bacterial pathogen. In fact, this work is part of the continuum in a typical systems biology work flow.

## Conclusion

The RNA-Seq based transcriptome map of *M. haemolytica *PHL213 validated annotated open reading frames and led to the discovery of potential novel protein coding regions. We identified operon structures and were able to fix exiting annotation errors by correcting gene boundaries. The availability of experimentally validated open reading frames, potential novel sRNA, potential protein coding regions, and operon structures form the basis for future investigations to determine the role of these elements during BRD pathogenesis. This study also demonstrates the utility of free and easy to bioinformatics tools for RNA-Seq data analysis workflow.

## List of abbreviations used

BAM: Binary Alignment/Map; BLAST: Basic Local Alignment Search Tool; BRD: Bovine Respiratory Disease; BHI: Brain Heart Infusion; DOOR: Database for prOkaryotic OpeRons; EIR: Expressed Intergenic Region; GLIMMER: Gene Locator and Interpolated Markov ModelER; MAQ: Mapping and Assembly with Qualities; ORF: Open Reading Frame; PPP: Prokaryotic Promoter Prediction; Rfam: RNA families; RNAP: RNA Polymerase; SAGE: Serial Analysis of Gene Expression; SAM: Sequence Alignment/Map; SOAP: Short Oligonucleotide Analysis Package; sRNA: small RNA; sRNAdb: small non-coding RNA database.

## Competing interests

One of the authors, James M Watt, is currently employed with Eagle Applied Science. Since the research work for this manuscript was performed when he was an employee at College of Veterinary Medicine, Mississippi State, Mississippi, it does not alter the authors' adherence to all the BMC Bioinformatics policies on sharing data and materials. The authors declare that they have no competing interests.

## Authors' contributions

JSR developed the analysis workflow with RK and BN, wrote all scripts required for analysis, carried out data analysis, and wrote the initial draft of this manuscript. JMW prepared the RNA for conducting RNA-Seq. SCB, MLL, and BN conceived and designed this collaborative study, and helped with data analysis and interpretation. BN helped draft the final version of the manuscript. All authors read and approved the final manuscript.

## Supplementary Material

Additional file 1 **Complete description of suggested corrections to existing annotation, and identified frameshift mutations**. Sheet 1 labeled 'Annotation Errors' contains corrections to annotated genes in *M. haemolytica *PHL213, previously annotated gene locus and length, suggested correction to its locus. RNA-Seq expression based observed gene and protein length along with description of the exception in the genome that led to the annotation error; in case of mutated start, the mutated amino acid; BLASTX hit used to correct the annotation and its description. Sheet 2 labeled 'Frameshift' contains the two frameshift mutations identified, their frame locus and the BLASTX hit used to identify the frameshift. Click here for file

Additional file 2 **RNA-Seq based expression profile of annotated genes**. The sheet labeled as 'MH_Expressed' consists of annotated genes identified as expressed in the RNA-Seq experiment, the observed coverage, average reads per base for each gene and the description of the gene, Sheet 2 labeled as 'MH_NotExpressed' contains annotated genes identified as not expressed in RNA-Seq experiment, the observed coverage, average reads per base for each gene and the description of the gene. Click here for file

Additional file 3 **Comparison of co-expressed gene pairs identified by RNA-Seq and operons predicted by DOOR**. Sheet 1 labeled 'MH_DOOR' has a list of operons predicted by DOOR. Sheet 2 labeled 'MH_DOOR_Pairwise' contains a list of co-expressed gene pairs predicted by DOOR. Sheet 3 labeled 'MH_JR' contains a list of operons identified in our RNA-Seq experiment. Sheet 4 labeled 'MH_JR_Pairs' contains a list of co-expressed gene pairs identified by RNA-Seq. Sheet 6 labeled 'MH_Pairwise_Common' contains a list of co-expressed gene pairs common to both DOOR and RNA-Seq. Sheet 7 labeled 'MH_DOOR_Unique' contains a list of co-expressed gene pairs unique to DOOR. Sheet 8 labeled 'MH_JR_Unique' contains a list of co-expressed gene pairs unique to our RNA-Seq experiment. Click here for file

Additional file 4 **Results of *M. haemolytica *PHL213 sRNA searched against sRNAdb**. Putative sRNA identified by RNA-Seq were searched against other bacterial sRNA in small non-coding RNA database (sRNAdb) by conducting BLASTN searches. Click here for file
